# What is the rhythm?

**DOI:** 10.1007/s12471-018-1154-9

**Published:** 2018-08-31

**Authors:** A. W. G. J. Oomen, R. W. Sy

**Affiliations:** 1 Department of Cardiology, Royal Prince Alfred Hospital, Camperdown, NSW Australia; 2 Sydney Medical School, University of Sydney, Sydney, NSW Australia

A 22-year-old female presented with a history of palpitations. During follow-up the electrocardiogram (ECG) shown below (Fig. 1) was recorded. Her baseline ECG during sinus rhythm showed no pre-excitation. Physical examination and echocardiogram were both unremarkable.

**Fig. 1:**
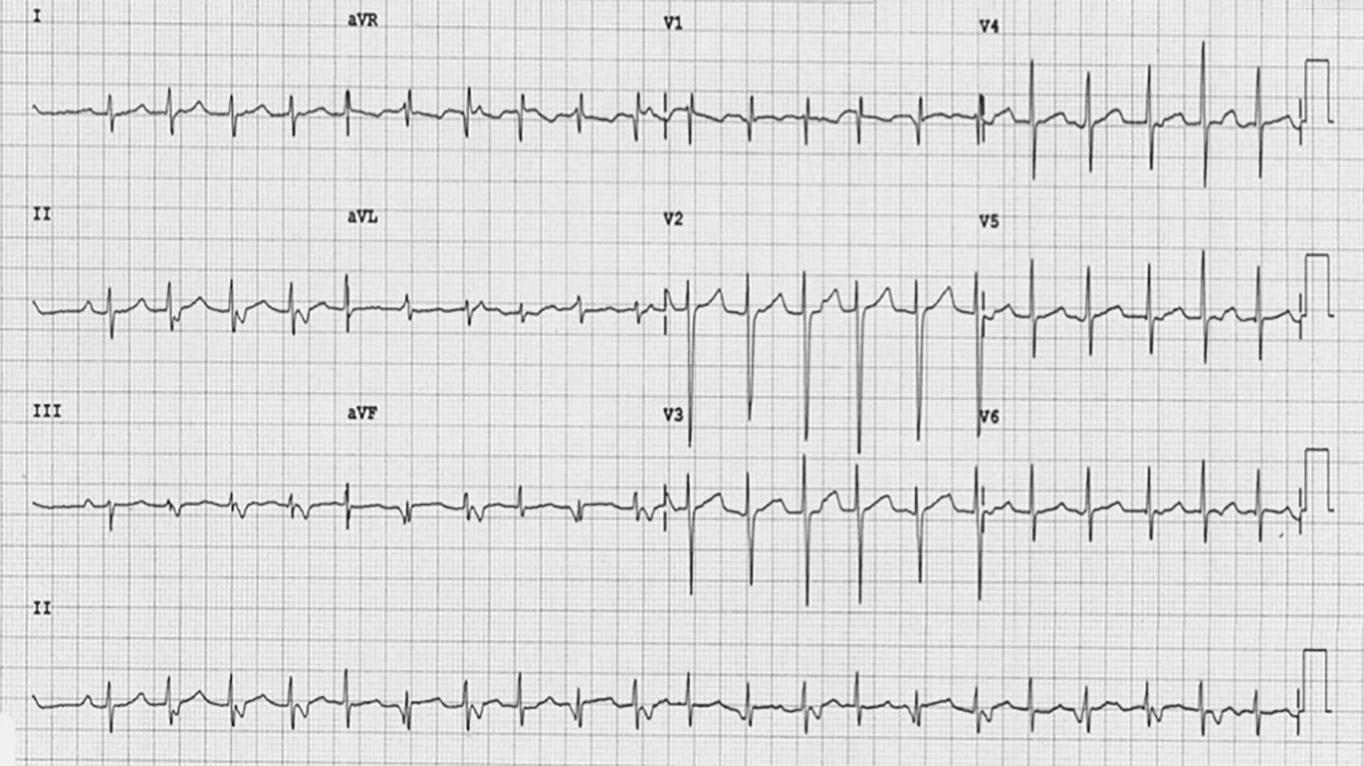
ECG at presentation

What is the most likely diagnosis for the arrhythmia?

## Answer

You will find the answer elsewhere in this issue.

